# The Impact of Mild COVID-19 on Executive Functioning and Mental Health Outcomes in Young Adults

**DOI:** 10.3390/healthcare10101891

**Published:** 2022-09-28

**Authors:** Piruza Manukyan, Alena Deviaterikova, Boris B. Velichkovsky, Vladimir Kasatkin

**Affiliations:** 1 Research Institute for Brain Development and Peak Performance, Peoples’ Friendship University of Russia, 117198 Moscow, Russia; 2 Faculty of Psychology, Lomonosov Moscow State University, 125009 Moscow, Russia; 3 Cognitive Foundations of Communication Laboratory, Moscow State Linguistic University, 119034 Moscow, Russia; 4 Department of General Psychology, Moscow Institute of Psychoanalysis, 119334 Moscow, Russia

**Keywords:** executive functions, COVID-19, long COVID, depression, anxiety, fatigue

## Abstract

COVID-19 is associated with a range of long-lasting symptoms related to cognitive and psycho-emotional spheres. Even mild cases of the disease can lead to persistent cognitive deficits and deterioration of the psycho-emotional state. The purpose of our study was to examine the presence and frequency of psycho-emotional disorders and cognitive deficits in students who recovered from mild form of COVID-19. A total of 40 COVID-19 survivors and 25 healthy controls participated in our study. We assessed three core cognitive functions (inhibition, working memory, task-switching), reaction time and motor speed. We also assessed depression, anxiety and fatigue with self-report questionnaires. The COVID-19 group manifested increased depression and decreased inhibition in comparison with the controls. Our results show that even in young adults who have recovered from mild COVID-19, there are persisting cognitive and psycho-emotional deficits.

## 1. Introduction

In 2019, the whole world faced a new virus, SARS-CoV-2, and the disease it causes was officially named Coronavirus Disease 2019 (COVID-19) by the World Health Organization (WHO) [1]. The massive and rapid growth in number of infected cases has led to government measures to limit the spread of the disease by introducing various forms of isolation [2]. It was necessary to cope with the huge flow of cases, while it was not clear what delayed effects the virus carries, both on the somatic health of the population and the psychological well-being [3]. COVID-19 is associated with a range of symptoms related to different systems: respiratory, cardiovascular, immune, endocrine, nervous, etc. [4,5]. As of August 2022, World Health Organization reported more than 590 million confirmed COVID-19 cases [6] and given the effect of the virus on the nervous system [7], cognitive and emotional spheres are shown to be particularly vulnerable to the infection [8,9]. By now a lot of studies on neurocognitive symptoms after the SARS-CoV-2 infection indicate that cognitive deficits and emotional distress are quite common among people who survived COVID-19 [10,11,12], but its underlying pathophysiology remains unknown. One of the potential mechanisms is inflammatory response that can affect cognitive functions through disruption of cerebral vasculature integrity, neurotransmission, and loss in processes of neurogenesis [13]. Inflammation is shown to be a risk factor for long-term cognitive deficits [14]. The other potential way through which the virus can affect the nervous system (hence the cognition) is related to the olfactory system [15].

Another problematic issue is how the illness affects younger populations. The first observations showed that young adults easily tolerate the virus itself and do not carry any serious consequences of the disease [16]. However, an increase in infected cases has led to reports on cognitive deficits among young adults (memory, attention, difficulty thinking) [12,17], as well as the deterioration of the psycho-emotional state (depression, fatigue, anxiety, etc.) [18,19]. Moreover, there are such complaints among those patients who needed [20], and among those who did not need hospitalization and medical assistance. By now, cognitive deficits are observed in 50–60% of patients who have been infected, regardless of the severity of the disease itself and the accompanying diseases [21]. Cognitive and psycho-emotional disorders may potentially be related to each other: patients who had cognitive deficits also had mood disorders [22]. The psycho-emotional disorders of patients are not homogeneous: the symptoms of PTSD disappear about 3 months after the end of the disease, unlike the symptoms of depression, which can persist much longer [21]. Persistent cognitive deficits can play a similar detrimental role in the deterioration of the quality of life of COVID-19 survivors, in the self-reinforcing circle of depressive psychopathology, neurocognitive disorders and a decrease in overall functioning.

The vulnerability of the nervous system to infection requires to continue the research on the effects of COVID-19 on different populations with validated objective tools. Among young adults receiving higher education, cognitive and emotional decline can lead to a deterioration in their academic performance and a decrease in the quality of their education. Thus, the main aim of the present study is to examine whether young people who have recovered from mild COVID-19 have persisting cognitive deficits and symptoms related to emotional wellbeing.

## 2. Materials and Methods

Participants for this study were recruited as part of the larger research project “Improvement of cognitive functions in RUDN university students with post-COVID syndrome and other neurocognitive dysfunctions”, which aimed at studying the relationship between neurocognitive dysfunctions in people who have recovered from COVID-19 and metabolic abnormalities and deterioration of fundamental motor skills. The study was approved by the Medical Institute Ethics Committee of RUDN University. In order to recruit the participants, we sent invitation letters; in addition, the coordinator of the study attended lectures at the university and invited the students to participate in the study free of charge. All participants were provided with detailed information about the study and signed an informed consent form. The information on COVID-19 experience, presence of post-COVID-19 syndrome and corresponding symptoms according to students’ self-report was documented during a conversation with them. The study was conducted between February 2022 and June 2022.

A total of 40 students (29 females and 11 males) of RUDN University aged between 17 and 27 who had COVID-19 and 25 age-matched controls (20 females and 5 males) were recruited. We included patients with mild COVID-19, who had polymerase chain reaction (PCR) confirmed infection of SARS-CoV-2 at least three months prior to the assessment procedure. All patients within COVID-19 group included in our study did not receive intensive care during COVID-19 and recovered without any complications or medical assistance. Inclusion criteria also included: (a) no prior or current neurological disorders due to which cognition could be impaired, (b) normal or corrected to normal vision. To assess the presence and severity of cognitive and psycho-emotional symptoms in students who suffered from COVID-19, the participants who had positive PCR results were matched with the controls who had never tested positive for SARS-CoV-2 before and reported they never had any of the COVID-19 symptoms. Basic information about two groups is shown in Table 1.

### 2.1. Assessment Procedure

At the first stage of the study, we conducted interviews with the participants to identify the complaints after COVID-19. For the controls, this part was not applicable. Further, all participants underwent an assessment of cognitive functions with quantitative computerized tests and mental health assessment with self-report questionnaires. We performed mental health status assessment with hospital anxiety and depression scale (HADS), aimed at identifying and assessing the severity of anxiety and depression [23], fatigue rating scale (FAS) aimed at identifying and assessing the severity of fatigue (De Vries et al., 2004). For executive functions (EF) assessment, we chose tasks aimed at assessing three basic executive functions adapted from Miyake and colleagues [24]: inhibition, working memory updating and task-switching. We also used a choice reaction test from the computer-based Vienna System to assess reaction time and motor speed [25].

#### 2.1.1. Inhibition

To assess inhibitory control, we used the Go/No Go paradigm. The task consisted of a target stimulus or “Go” stimulus (the letter X) and distractors or “No Go” stimuli (14 other letters). The ratio of target and distractor presentations was 4 to 1, and the stimuli were presented in a random way. The participants needed to press the space bar every time they saw the target and not to react to distractor stimuli. The stimuli were being presented for 300 ms, and the interval between stimuli was 700 ms. Before the main test, which consisted of 200 trials, the participants performed training series with 20 trials. The registered outcomes were reaction time and number of errors.

#### 2.1.2. Working Memory Updating

The n-back paradigm is usually used to assess working memory updating [26]. In the task participants were presented with a sequence of digits and needed to determine whether the presented digit was the same as n trials before (in our study, we used 2-back version) and press the corresponding button. Before the main test trials (62 digits with 15 matching events) participants performed practice trials (10 digits with 3 matching events). The registered outcomes were number of errors and average reaction time.

#### 2.1.3. Task-Switching

To assess task-switching performance, we used the Number–Letter task [27]. In this task, a pair of one number and letter appears in sequential order on one of the four quadrants. When the stimulus appeared in one of the two upper quadrants, the participants needed to indicate whether the number was odd or even by pressing the corresponding button. When the stimulus appeared in one of the bottom quadrants it was necessary to indicate whether the letter was a vowel or a consonant. The stimuli were being presented until the button was pressed. Before the main series with 128 trials, the participants performed training series consisting of 24 trials.

#### 2.1.4. Choice Reaction Test

We used the choice reaction test from the computerized Vienna Test System (Schuhfried, RT, test form S5) to assess reaction time and motor speed. The participants needed to react to target stimuli as fast as possible. The target stimuli were either yellow plus red circles presented together or yellow circle presented with the sound. All other cases needed to be ignored. The registered outcomes were reaction time, motor speed in milliseconds and number of correct responses.

### 2.2. Data Analysis

For the statistical analysis of differences in cognitive performance and psycho-emotional state indicators between the COVID-19 group and the controls, the Mann–Whitney *U* test was used as a nonparametric analogue of the *t*-test as the data did not follow a normal distribution. A significance level was set at 0.05.

## 3. Results

All 65 participants successfully went through the computerized tasks procedure aimed at assessing executive functions, but some participants did not complete the choice reaction task due to lack of time. For the questionnaires, there were several unfinished and partially completed ones, so we had to exclude them from the analysis. The information on missing data by group is presented in Table 2.

The results of the statistical analysis of cognitive performance and mental health indicators are shown in Table 3. The results of the mental health indicators are presented first, followed by the results of cognitive performance on four tasks.

There were no significant differences on HADS anxiety subscale between two groups. Participant in COVID-19 group scored significantly higher in the HADS depression subscale (*p* = 0.003). The groups did not also significantly differ in fatigue (FAS) (*p* = 0.051). For the HADS depression subscale 8.3% of the participants in control group and 25.7% of the participant in COVID-19 group scored greater than 10 (≥11 is considered as moderate or severe case). In total, 20.8% of the participants in control group and 20% of the participant in COVID-19 group scored greater than 10 for the HADS anxiety subscale. For fatigue, 56.5% of the participants in control group and 80% of the participants in COVID-19 group scored greater than 21 (≥22 indicates fatigue).

There were no significant differences between COVID-19 group and the controls in the N-back task neither on reaction time (RT) nor on number of errors.

For the Go/No Go task, participants from the COVID-19 group scored significantly higher in number of errors (*p* = 0.033). RT in the Go/No Go task was not different between the two groups.

For the switching task, the groups did not differ in number of errors during both repetition and switching conditions (including switching cost in number of errors). There was also no difference in RT during both conditions (including switching cost).

In the choice reaction tasks, there was no difference between two groups in all three parameters: RT, motor speed and accuracy.

## 4. Discussion

In this study, we examined the presence and frequency of fatigue, depression, anxiety and cognitive dysfunctions (mostly focusing on three core executive functions) in young people after mild COVID-19.

The results of our study regarding the emotional wellbeing of people recovered from COVID-19 showed that people in COVID-19 group scored significantly higher in the depression subscale than the controls indicating that depression is among. This is consistent with other studies that reported similar results [21,22,28]. High rates of depression among students can seriously reduce their academic performance and therefore affect their quality of life. Additional measures are needed to reduce the level of depression among students. In particular, physical activity can be beneficial, since it is shown that physical activity can reduce symptoms of depression [29,30].

In our study, the groups did not significantly differ in scores of anxiety and fatigue. In other studies also conducted on students, various data were obtained on the level of anxiety among students, depending on their cultural affiliation. Perhaps the level of anxiety is related to how effective the measures to combat the pandemic have been taken internally and how protected the students feel [31].

It is interesting to note that the majority of cases in both groups showed the presence of fatigue—in 56.5% of the controls and in 80% of the participants affected by COVID-19—which could be related to the intense academic period (given that most of the participants of our study were students) [32]. One of the means of combating the pandemic has become social isolation and online learning, which, according to research, leads to greater psychological stress and fatigue among students than the traditional format of education [33,34].

Another main finding of our study was that the group of participants affected by COVID-19 showed worse performance on inhibition task. For the performance of other cognitive tasks, the groups did not significantly differ. This suggests that even in mild types of COVID-19, there could be slight impairments in cognition after the recovery. These results are consistent with existing studies showing cognitive deficits in people affected by COVID-19 [35,36]. By now, the presence of long-lasting cognitive symptoms is evident, but the question of underlying mechanisms remains open: it is not clear both the cause of the pathological process itself and which functions are most susceptible to impairments [37]. The results of our study related to the cognitive sphere may somehow indicate the dynamics of the recovery process on a neurobiological level; in particular, deficits in inhibition may be related to the fact that brain regions involved in inhibitory processes recover relatively slower. However, the results must be interpreted cautiously due to the study limitations.

The main limitations of the present study were the small overall sample size and the smaller sample size of the control group relative to the group affected by COVID-19 due to the difficulties of identifying people who did not have COVID-19. These limitations, in turn, restricted our capacity to do more advanced statistical analysis. Further research on much larger cases is needed. One more limitation is the participants’ young age, which restricts the generalization of the results to the population, though the aim of our study was to examine whether the youth would have persisting neurocognitive symptoms after COVID-19. We also included only mild cases of COVID-19, so the results should not be generalized to more severe cases, and our sample consisted of young people studying in Russia; thus, further studies on students living in other countries are required to make more general conclusions.

## 5. Conclusions

This study found that there is a statistically significant difference between young people after mild COVID-19 and the controls in cognitive functioning and psycho-emotional sphere. Individuals from COVID-19 group have higher rates of depression and worse performance on inhibition task. Thus, young adults who have recovered from mild COVID-19 may also face cognitive impairments and symptoms of depression. Given the importance of the students’ well-being, it is necessary to pay attention to the difficulties they face after recovering from COVID-19 as it can have a negative impact on their academic performance. It may be necessary to create special rehabilitation programs to help students who have had COVID-19. In summary, despite all the limitations, the results of the study contribute to the growing amount of research on the long-lasting consequences of COVID-19.

## Figures and Tables

**Table 1:** Demographic characteristics by group.

	COVID Group (*n* = 40)	Controls (*n* = 25)	Total (*n* = 65)
age			
mean (SD)	19.9 (2.06)	20.32 (2.91)	20.06 (2.4)
range	18–27	17–26	17–27
sex			
female	29 (72.5%)	20 (80%)	49 (75.4%)
male	11 (27.5%)	5 (20%)	16 (24.6%)

**Table 2:** Information on missing data by group.

Assessment Tool	COVID Group		Controls	
	**Completed (*n*)**	**Missing (*n*)**	**Completed (*n*)**	**Missing (*n*)**
HADS	35	5	24	1
FAS	35	5	23	2
Go/No Go	40	0	25	0
N-back	40	0	25	0
Task-switching	40	0	25	0
Choice reaction	39	1	22	3

**Table 3:** Descriptive statistics and Mann–Whitney *U* test results for two groups.

Assessment Tool	COVID Group				Controls				Mann–Whitney *U*	*p*-Values
	*n*	Mean	Median	SD	*n*	Mean	Median	SD		
**HADS ^1^**	35				24					
**Depression**		**7.48**	**7**	**4.59**		**4.20**	**3**	**4.23**	**229.5**	***p* = 0.003**
**Anxiety**		7.82	8	3.85		7.12	7	3.57	373	*p* = 0.471
**FAS ^2^**		27.06	26	7.50		23.26	22	7.11	279.5	*p* = 0.051
**Go/No Go**	40				25					
**RT ^3^**		0.35	0.34	0.04		0.35	0.36	0.02	596.5	*p* = 0.193
**Nerr**		**10.17**	**9**	**5.71**		**7.52**	**6**	**6.50**	**342**	***p* = 0.033**
**N-back**	40				25					
**RT**		1.13	0.81	0.89		1.17	0.94	0.73	542	*p* = 0.576
**Nerr**		12.92	7.5	12.91		18.60	7	27.81	489	*p* = 0.887
**Task-switching**	40				25					
**No switch RT (seconds)**		1.25	1.05	0.77		1.26	1.21	0.52	569.5	*p* = 0.352
**No switch Nerr ^4^**		12.77	2.5	17.03		12.40	3	16.67	509	*p* = 0.908
**Switch RT (seconds)**		2.27	2.01	1.10		2.35	2.38	0.91	541.5	*p* = 0.580
**Switch Nerr**		13.47	4.5	15.75		13.56	8	15.95	526	*p* = 0.73
**Switch cost RT**		1.02	0.90	0.75		1.09	1.03	0.70	547	*p* = 531
**Switch cost Nerr**		0.70	1	2.54		1.16	0	2.60	518.5	*p* = 0.806
**Choice reaction**	39				22					
**Motor Speed**		577.82	569.00	93.85		541.45	526.00	59.37	393.5	*p* = 0.599
**RT (milliseconds)**		198.36	187.00	60.41		191.41	181.00	65.65	313.5	*p* = 0.084
**Number of CR ^5^**		15.72	16	0.51		15.64	16.00	0.49	387	*p* = 0.432

^1^ Hospital Anxiety and Depression Scale, ^2^ Fatigue Assessment Scale, ^3^ Reaction time, ^4^ Number of errors, ^5^ Correct responses.

## Data Availability

All the data are available upon reasonable request to the authors.
